# Prevention of Hepatitis C in Women^1^

**DOI:** 10.3201/eid1011.040624_04

**Published:** 2004-11

**Authors:** Beth P. Bell, Eric E. Mast, Norah Terrault, Yvan J.F. Hutin

**Affiliations:** * Centers for Disease Control and Prevention, Atlanta, Georgia, USA;; † University of California at San Francisco, San Francisco, California, USA;; ‡ World Health Organization, Geneva, Switzerland

**Keywords:** hepatitis C, hepatitis C infection, women

Hepatitis C is a major public health problem in the United States. Although the incidence of new infections declined substantially in the past decade, approximately 25,000 persons are infected each year. In total, an estimated 2.7 million Americans have chronic hepatitis C virus (HCV) infection and are at risk for HCV-related chronic liver disease and hepatocellular carcinoma (HCC).

The most common exposure associated with HCV infection is use of injection drugs. Other less commonly identified risk factors include sexual contact; transfusions before blood screening was implemented; and occupational, nosocomial, and perinatal exposures. Although sources of HCV infection are the same for men and women, the overall prevalence of HCV infection is lower among women than men, which is likely related to the lower prevalence of injection-drug use among women.

The risk for HCV transmission from mother to infant is about 5%–6%; transmission occurs only from women who are HCV RNA positive and is higher among those coinfected with HIV (≈18.7%) than among women not infected with HIV (≈5.4%). The influence of factors such as maternal viral titer and interventions at the time of delivery is unclear. Studies indicate that breastfeeding is not a risk factor for perinatal transmission.

Most hepatitis C prevention strategies are gender neutral and include screening and testing donors of blood, plasma, organ, tissue, and semen; virus inactivation of plasma-derived products; effective infection control practices; identification, counseling, and testing of at-risk persons; and medical management of infected persons. Pregnant women with risk factors for infection should be identified, screened, and counseled regarding the risk for perinatal transmission.

## Clinical Reports

Although risk factors for HCV acquisition are similar among men and women, women are at higher risk of acquiring HCV from sexual contact with an HCV-infected partner and more likely to be initiated into drug use, share needles, or be injected by a sexual partner. Among HCV-infected women, pregnancy may lead to worsening of histologic disease. Other gender differences in the natural history of hepatitis C are that the rate of spontaneous HCV clearance may be higher among women than men, the risk for fibrosis progression and HCC are lower in women than men, and alcohol use by women with hepatitis C is likely to have more pronounced negative effects on the liver than is observed among HCV-infected men. There do not appear to be substantial gender differences in response to currently available therapy.

## Hepatitis C Epidemiology and Prevention: the International Perspective

Approximately 2.2% of the world's population, 130 million people, are infected with HCV. Worldwide, an estimated 325,000 deaths from HCV-attributable HCC and cirrhosis occur annually. In industrialized countries, most HCV-infected persons have prevalent, chronic infections, attributable to past exposures such as injection drug use, blood transfusions, and sexual contact. Primary prevention strategies include reducing harm and preventing nosocomial transmission. In developing countries, many incident, new infections are due to health care-related exposures such as unsafe injections, and prevention strategies focus on safe health care as well as reducing harm.

